# No association between incidence of type 1 diabetes and rotavirus vaccination in Swedish children

**DOI:** 10.3389/fimmu.2023.1175071

**Published:** 2023-08-10

**Authors:** Amanda Rangert, Carin Oldin, Marie Golsäter, Johnny Ludvigsson, Karin Åkesson

**Affiliations:** ^1^ Division of Pediatrics, Department of Biomedical and Clinical Sciences, Linköping University, Linköping, Sweden; ^2^ Futurum – Academy of Health and Care, Region Jönköping County, Jönköping, Sweden; ^3^ Child Health Services, Jönköping, Region Jönköping County, Jönköping, Sweden; ^4^ CHILD - Research Group, School of Health and Welfare, Jönköping University, Jönköping, Sweden; ^5^ Crown Princess Victoria Children´s Hospital, Linköping, Sweden; ^6^ Department of Pediatrics, Ryhov County Hospital, Jönköping, Sweden

**Keywords:** type 1 diabetes, children, rotavirus (RV), vaccination, incidence

## Abstract

**Background:**

Rotavirus infection is a potential trigger of type 1 diabetes (T1D) and rotavirus vaccination is hypothesized to decrease the incidence of T1D. In Sweden, rotavirus vaccination was introduced in 2014 in two regions and from 2019, nationwide. This study aims to investigate the association between rotavirus vaccination and incidence of T1D in Swedish children and whether rotavirus vaccination is associated with a change in clinical manifestation at diabetes onset.

**Methods:**

A nationwide register-based study with all Swedish children <15 years of age, diagnosed with T1D 2009-2019 was conducted. 7893 children were retrieved. Nationwide vaccine coverage was collected from Child Health Services. Three vaccine groups were created: I: Vaccination start 2014; II: Gradual vaccination start 2016-2018; III: No vaccination. Incidence rates of T1D before (2009-2014) and after (2014-2019) introduction of rotavirus vaccine were compared.

**Findings:**

The mean incidence of T1D in children <15 years was 42·61 per 100 000 during the observed period. When comparing the years before and after 2014 the incidence rate ratio (IRR) for children <5 years was 0·86 in group I (p=0·10), 0·85 (p=0·05) in group II and 0·87 (p=0·06) in group III. A similar IRR reduction was also seen among older children who received no vaccine. Children developing or not developing T1D were vaccinated to the same extent. No differences regarding clinical manifestation at onset associated with rotavirus vaccination were seen.

**Interpretation:**

There is no association between rotavirus vaccination in children and incidence or clinical manifestation of T1D.

## 1 Introduction

Type 1 diabetes (T1D) is regarded to be an autoimmune disease where pancreatic islet β-cells, producing insulin, are destroyed (1, 2). It is the most common autoimmune disease among children (2) and the incidence has increased in Sweden during several decades (3, 4). The epidemiology of T1D varies greatly around the world and Sweden has, next to Finland, the highest incidence worldwide (5). Many potential environmental factors, such as viral infections, psychological stress, diet, birthweight, and infant growth, have been suggested to contribute to the development of the disease (6). Among the environmental factors, viral infections may be considered the most evidence-based. Different enteroviruses may be associated with T1D whereof one of the potential viruses is the rotavirus (7–9).

Rotavirus is a virus causing gastroenteritis in young children. It is the most common cause of severe gastroenteritis among children under five years of age (10). Reinfections do occur, but usually, the intensity of the disease decreases for each repeated infection (11). The infection mainly affects children between 6-24 months of age and was estimated to be responsible for ca 0.5 million deaths worldwide each year before the vaccination was started (10, 12).

It has been suggested that enterovirus infections can both precipitate the autoimmune reactions and increase the risk of islet autoimmunity progressing to overt T1D (13). A potential linkage between T1D and specifically rotavirus was suggested more than 20 years ago when a study showed a significant similarity between the autoantigens IA-2 and GAD and human rotavirus (14). This was further confirmed in another study, finding evidence indicative of molecular mimicry of T-cell epitopes in rotavirus and islet autoantigens (15). In mice, infection by rotavirus induces a decrease in pancreatic mass (16) and exacerbate autoimmune diabetes with an accelerated onset (17). In children, the islet autoimmunity might be caused or intensified by a rotavirus infection, which may implicate vaccination being protective (7, 18).

Findings about the association between T1D and rotavirus lead to considerations about vaccination against rotavirus and its potential effect on the incidence of T1D. Various studies have been done but with diverse results. In Australia the incidence of T1D before and after introduction of rotavirus vaccination was studied using public data. The results displayed an incidence decrease by 15% among children aged 0-4 years the year after introduction of vaccination. However, there was a similar change in incidence the same year also for older children aged 5-14 years, although not statistically significant (19). In the U.S another cohort study was done comparing vaccinated and unvaccinated children where the results showed a 41% reduction among the vaccinated children with the greatest reduction among infants (20). Other American studies showed no association between rotavirus vaccination and the incidence for T1D (21), celiac disease or autoimmune thyroiditis (22). A Finnish nationwide cohort study comparing the risk for T1D between rotavirus-vaccinated and unvaccinated children showed no difference (23), similar to results from a larger cohort study in the UK (24). A review summarizing the evidence considering the association of rotavirus with T1D concluded that the connection remains unclear and there is no clear evidence for either protection or increased risk for T1D because of rotavirus vaccination (25). This is in line with a recent meta-analysis, including several of the studies mentioned above, where researchers concluded no association between rotavirus vaccine and T1D or celiac disease (26).

Vaccination against rotavirus was introduced in 2014 in Sweden when two different regions, Stockholm and Jönköping, started offering vaccination to all infants. After this introduction of the vaccine in Sweden, hospital admission due to gastroenteritis among children aged 0-4 years decreased by 57% in the vaccinated area (10). Gradually, seven other regions introduced the rotavirus vaccination between 2016-2018 and since Sept 1^st^ 2019 the remaining 12 regions started vaccination as rotavirus vaccine became part of the Swedish national children immunization program (27). An oral vaccine with a two-dosed regimen is offered to all Swedish children at the age of six weeks and three months (27).

The differences in time of introduction of Rota-virus vaccination combined with the Swedish national registration of all newly-diagnosed children with T1D offers a possibility to compare the incidence of T1D in areas with or without rotavirus vaccination parallel in time.

### 1.1 Aim

The aim of this study was to investigate the association between rotavirus vaccination and incidence of T1D among Swedish children. We also wanted to analyze if rotavirus vaccination caused any changes in the clinical manifestation of the disease at onset.

## 2 Research in context

### 2.1 Evidence before this study

Repeated PubMed searches for relevant references was performed from November 2020 to April 2021, using the terms “rotavirus vaccination”, “rotavirus” and “type 1 diabetes”. Two previous studies from the U.S. and Australia had reported a significant decrease in T1D incidence rates among children vaccinated with rotavirus vaccine. A study in Finland and another study from the U.S showed no such effect, and a third study has just been published showing no effect on incidence of celiac disease or autoimmune thyroiditis. A recent review on rotavirus vaccine and T1D in humans, concluded that the association is unclear.

### 2.2 Added value of this study

Our large, nationwide study, which includes all children with T1D in Sweden 5 years before and after introduction of rotavirus vaccination shows no association between rotavirus vaccination and incidence of T1D in any age group, and no change of clinical manifestation of T1D.

### 2.3 Implications of all the available evidence

Rotavirus vaccination of infants worldwide is justified but for other reasons than preventing T1D.

## 3 Materials and methods

### 3.1 Study population

By using the SWEDIABKIDS database, all Swedish children under the age of 15 and diagnosed with T1D between 2009 and 2019 were identified (n = 7893). The pediatric part (SWEDIABKIDS) of the Swedish National Diabetes Register, is a national quality register and all patients have given informed consent to be registered (28). It includes data from 98% of Swedish children and adolescents with T1D, followed from diagnosis until turning 18 years of age. The coverage rate is based on the number of people, under the age of 18, who are registered in the National Diabetes Register in 2020 compared to people who in 2019 had at least one in- or outpatient visit with T1D (ICD E10) according to the National Patient Register. The Swedish National Patient Register includes all in-patient care and all outpatient doctor visits since 2001. The National Diabetes Register includes data on all types of diabetes, T1D, type 2 diabetes, other specified diabetes, and unspecified diabetes (codes E10, E11, E13, E14), according to WHO:s *International Classification of Diseases*, tenth version (ICD-10). Data on birthdate, sex and pH, occurrence of ketoacidosis, and age at diagnosis were collected.

To determine the incidence of T1D, statistics from Statistics Sweden was used to identify how many children were born and how many children under the age of 15 who live in each region in Sweden each year during 2009-2019. Statistics Sweden is responsible for official and state statistics in Sweden (29).

The chosen study population was stratified by year of diagnosis and incidence rates were calculated per 100 000 persons. To observe changes between the younger and the older children, three different age groups were created, from 0-4.9 years of age, from 5-9.9 years of age and from 10-14.9 years of age. To compare the regions, which started vaccination in 2014 with those who started gradually between 2016 and 2018 and those who did not offer vaccination during the studied period, three different groups were made. (**Appendix 3**) Incidence rates and clinical data such as pH and HbA1c before (2009-2014) and, for group I, after vaccination (2015-2019) were compared for all three groups.

### 3.2 Vaccination coverage

All children in Sweden are connected to a Child Health Service where they are offered vaccinations according to the national immunization program. The Regional Child Health Services in each region report their vaccination coverage to the national immunization register held at the Public Health Agency of Sweden. Data on vaccination coverage during 2014-2019 was obtained from the Head of Child Health services in the different regions.

The Head of Child Health services in region Stockholm, Jönköping (group I) and Västra Götaland (group II) collected individual vaccination status, through electronic access to the medical journal, of all children within the study population who had received rotavirus vaccination within the regional immunization program. With this data, a comparison between vaccination coverage for the children who later were diagnosed with T1D and the regional overall coverage was made.

### 3.3 Statistical analysis

The statistical analysis was conducted by IBM SPSS Statistics version 27. Descriptive statistics were used to analyze the study population. Continuous and normally distributed variables were presented as mean and standard deviation. Incidence rates were calculated per 100 000 persons with a 95% confidence interval (CI) according to Taylor series approximation of variance using OpenEpi Version 3 (30). Incidence rate ratios (IRR), with a 95% CI, were calculated to compare incidence rates between the years before (2009-2014) and the years after (2015-2019) introduction of the vaccine. Chi-squared test was used to compare categorical variables which were presented as proportions. For comparison of continuous values in the two different year spans independent samples T-test was used while one-way ANOVA was used to compare continuous values for three independent groups. The differences were regarded to be statistically significant at P < 0·05.

## 4 Results

### 4.1 Study population

A total of 7893 children under the age of 15 with T1D were retrieved from the SWEDIABKIDS register. 4529 children were boys (53·7%) and 3901 were girls (46·3%). 1784 (22·6%) of the children were 0-4·9 years of age, of these 967(54·2%) were boys and 817 (45·8%) girls. (**Appendix 5**).

### 4.2 Incidence rates - overall

**Figure 1:**
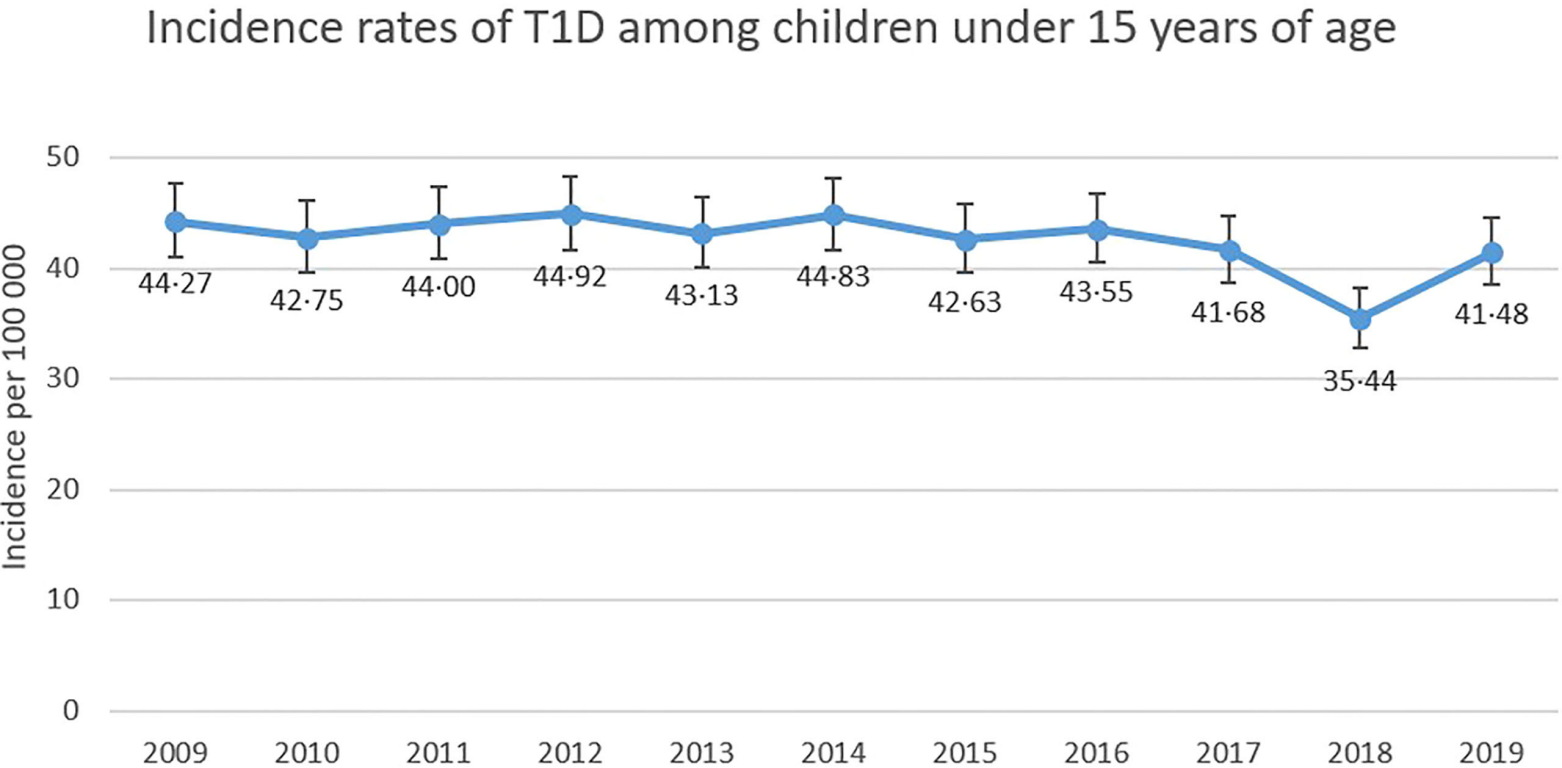
Incidence rates of T1D (with 95% CI) among Swedish children aged 0-14·9 years of age between 2009-2019.

**Figure 2:**
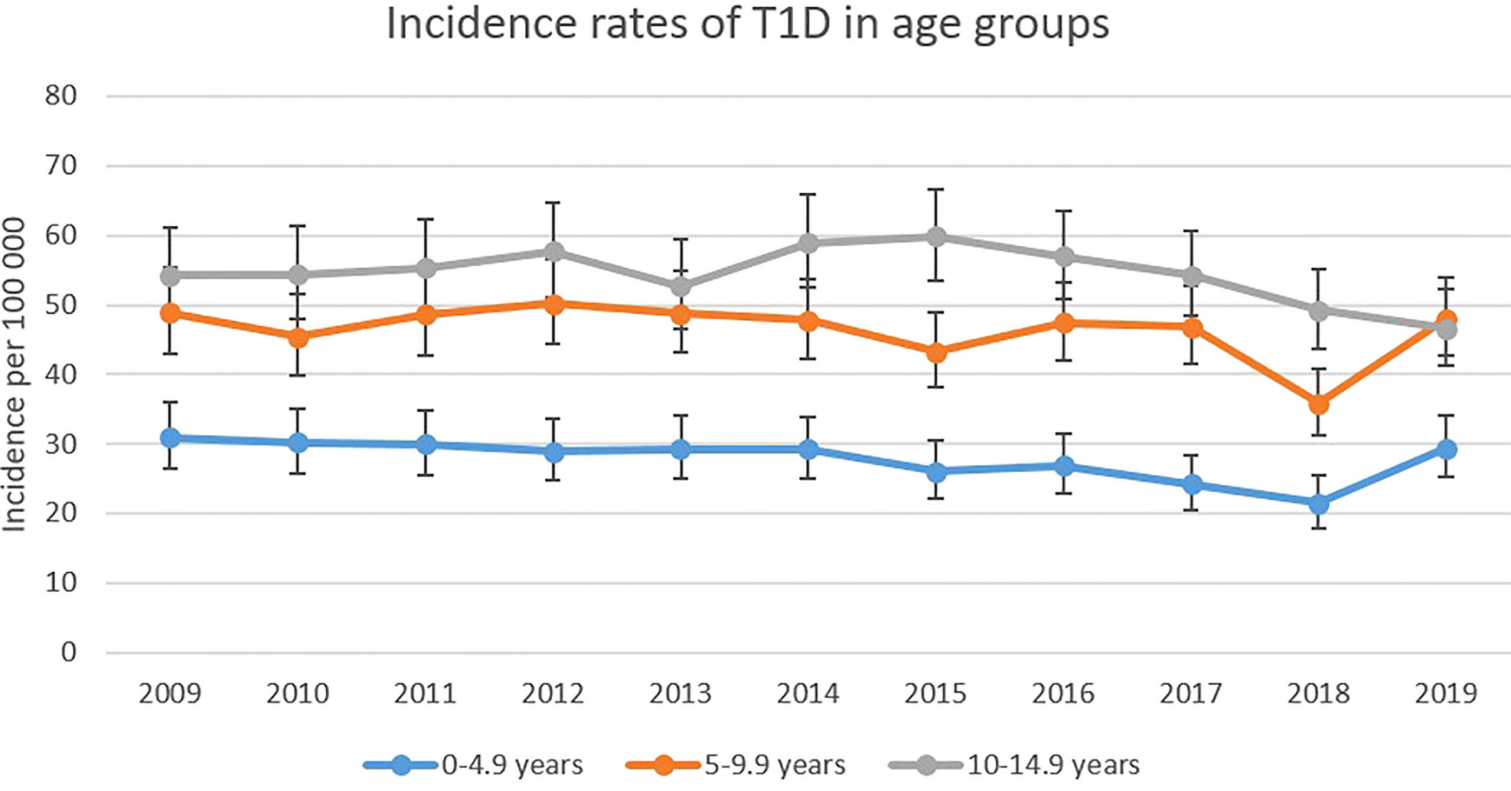
Age group-based incidence rates of T1D (with 95% CI) among Swedish children 2009-2019.

The incidence rate of T1D per 100 000 Swedish children <15 years of age between 2009 and 2019 was rather stable except for a decrease in year 2018 (35.44). The highest incidence was seen in 2012 (44·92) with a mean value during the study period of 42·61 cases per 100 000 persons (**Figure 1**). **Figure 2** displays the incidence of T1D in the three different age groups. Mean incidence rate was 27·91 per 100 000 persons for the youngest children, 46·45 per 100 000 for children aged 5-9·9 years and 54·55 per 100 000 persons for the oldest aged 10-14·9 years. In **Appendix 1** exact numbers of cases and children are presented.

### 4.3 Incidence rates - vaccination groups

**Figure 3:**
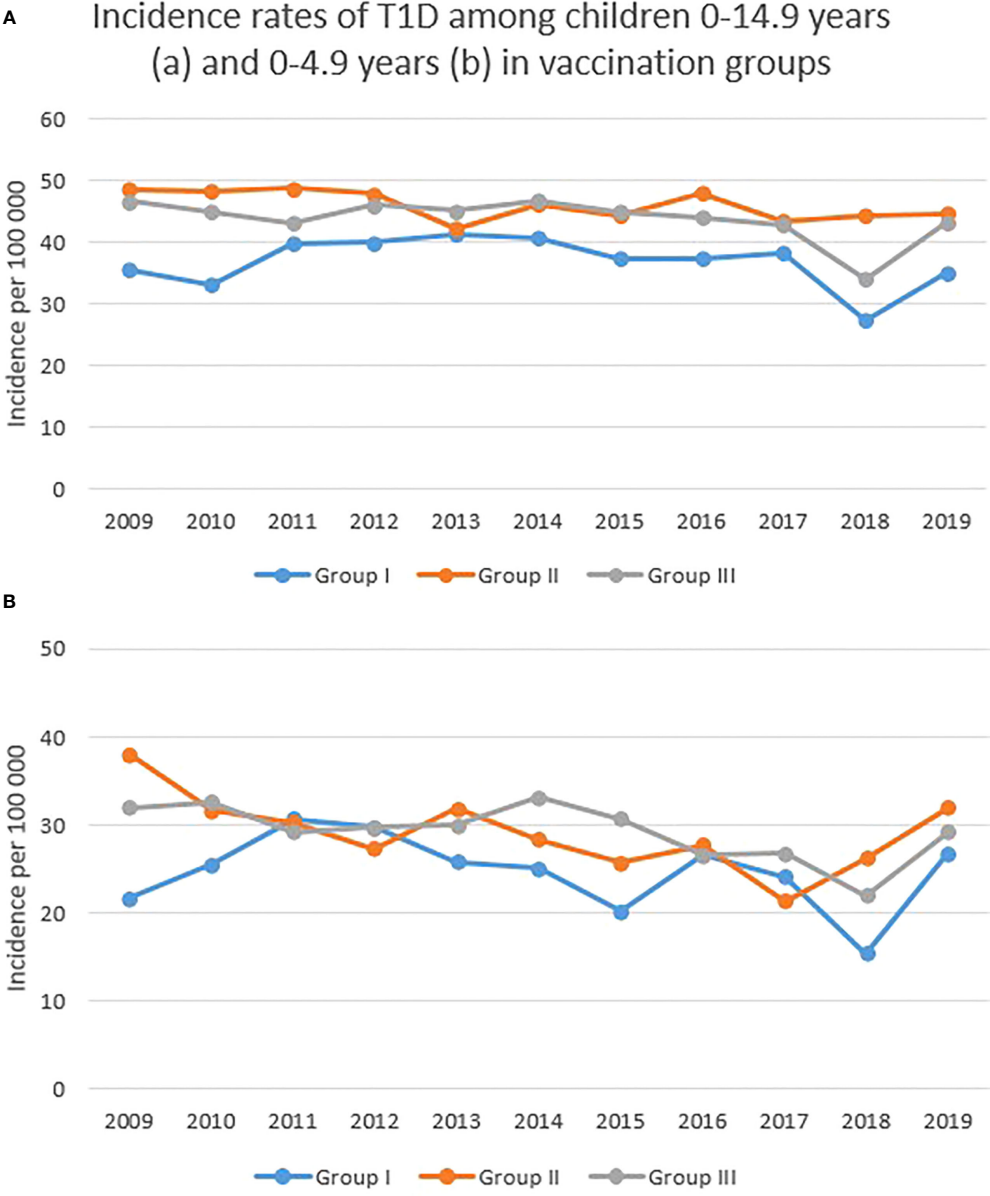
**(A, B)** Incidence rates of T1D among Swedish children aged 0-14·9 and 0-4·9 years of age depending on rotavirus vaccination status. Group I started rotavirus vaccination in 2014, group II started sometime between 2016 and 2018 and group III started in 2019.

Incidence rates for the three vaccination groups are shown in **Figure 3**. Group I had a lower incidence both before and after 2014. Group I had a mean incidence of 36·86 per 100 000 children, while the mean incidence for group II was 46·06 per 100 000 children and for group III it was 43·75 per 100 000 children between 2009 and 2019. **Figure 3B** shows the incidence rates for children aged 0-4·9 years of age in the three different vaccination groups. Mean rates were 24·69 for group I, 29·14 for group II and 29·25 cases per 100 000 children for group III during 2009-2019.

Average incidence rates per 100 000 persons, with a 95% CI, before and after introduction of rotavirus vaccine in Sweden are presented in **Table 1**. In group I, the earliest vaccine group, there was a decrease by 16% (IRR=0·84) among the oldest children 10-14·9 years (p=0·02), not offered rotavirus vaccine. Almost the same reduction (14%, IRR=0·86) was seen among the youngest children, thus the children who received the vaccination, but this reduction was not significant (p=0·10). There was no difference in incidence rate in age group 5-9·9 years in vaccine group I (IRR=0·98). A reduction of the same size as in group I was also seen in group III (IRR=0·87) among the youngest children (p=0·07) and the children aged 5-9·9 years (IRR= 0·85, p<0·01). This is the group that did not receive any vaccination. In group II with gradual introduction of rotavirus vaccination IRR was 0.85 (ns) among the youngest children and 0·98 (ns) among children aged 5-9·9 years and 10-14.9 years.

**Table 1:** Incidence rate ratios of T1D before and after introduction of rotavirus vaccine based on vaccination status and age-groups.

	Average rate per 100 000 persons (95% CI)			
	2009-2014	2015-2019	Incidence rate ratio (95% CI)	P value
Group I				
0-4·9 years	26·44 (23·31-29·86)	22·62 (19·52-26·06)	0·86 (0·71-1·03)	0·10
5-9·9 years	40·24 (36·19-44·61)	39·53 (35·40-44·00)	0·98 (0·85-1·14)	0·81
10-14·9 years	51·47 (46·58-56·74)	43·37 (38·89-48·21)	0·84 (0·73-0·97)	0·02
Group II				
0-4·9 years	31·22 (28·02-34·68)	26·58 (23·45-30·02)	0·85 (0·73-1·00)	0·05
5-9·9 years	50·72 (46·52-55·21)	49·59 (45·32-54·15)	0·98 (0·87-1·11)	0·72
0-14·9 years	60·25 (55·57-65·22)	58·91 (54·14-64·00)	0·98 (0·87-1·10)	0·70
Group III				
0-4·9 years	31·07 (28·14-34·22)	27·03 (24·13-30·18)	0·87 (0·75-1·01)	0·06
5-9·9 years	51·88 (48·00-56·00)	43·24 (39·61-47·13)	0·83 (0·74-0·94)	<0·01
10-14·9 years	54·27 (50·18-58·61)	55·17 (50·95-59·63)	1·02 (0·91-1·13)	0·77

Group I started rotavirus vaccination in 2014, group II started sometime between 2016 and 2018 and group III started in 2019.

### 4.4 Clinical manifestation

There was no difference in mean values of pH or HbA1c at diagnosis between the vaccine groups or any change of these values before and after vaccine introduction (**Table 2**) and no difference in incidence of keto-acidosis at onset between the three groups neither in 2009-2014 (p=0·56) nor 2015-2019 (p=0·67). Likewise, there was no significant difference in HbA1c between the groups neither before (ns) nor after vaccination (ns). Chi2 crosstab showed the proportions of diabetes ketoacidosis, defined as pH <7·30 (**Table 2**). Children in group I had, in both time periods, a somewhat higher proportion of ketoacidosis but there was no significant difference in ketoacidosis proportions between the vaccine groups in 2009-2014 (p=0·15) or in 2015-2019 (p=0·99). In addition, there was no difference in proportions before and after vaccine introduction in any of the groups (group I p=0·60, group II p=0·99, group III p=0·19).

**Table 2:** Clinical manifestation of T1D measured as pH and HbA1c at disease onset depending on vaccine group among children aged 0-4·9 years.

	2009-2014				2015-2019			
pH	Mean value (SD)	N	Missing	Proportion of diabetes ketoacidosis	Mean value (SD)	N	Missing	Proportion of diabetes ketoacidosis
Group I	7·34 (·10)	205	54	21·5%	7·34 (·11)	141	50	19·1%
Group II	7·35 (·11)	282	35	18·4%	7·34 (·11)	227	19	18·5%
Group III	7·35 (·09)	383	58	15·1%	7·35 (·11)	284	46	19·0%

HbA1c	Mean value (SD)	N	Missing		Mean value (SD)	N	Missing	
Group I	80 (19)	195	64		83 (19)	137	54	
Group II	79 (17)	279	38		80 (19)	227	19	
Group III	79 (19)	384	57		81 (19)	279	51	

Group I started rotavirus vaccination in 2014, group II started sometime between 2016 and 2018 and group III started in 2019.

For HbA1c there was no differences in any of the groups before and after 2014. (group I p=0·82, group II p=0·22, group III p=0·51).

### 4.5 Vaccination coverage

For a total of 125 children, later developing T1D, in region Jönköping, Stockholm and Västra Götaland we could collect individual vaccination status. Of these children, 91 received the rotavirus vaccine (Jönköping: 21/29, Stockholm 45/65, Västra Götaland 25/31). Regions Jönköping and Västra Götaland used the Rotarix vaccine, given in two doses, and region Stockholm used both Rotarix and Rotateq vaccine which is given in three doses. In region Stockholm three of the vaccinated children (3/45) only received two out of three Rotateq vaccine doses. Overall coverage of rotavirus vaccine in region Jönköping was varying between 76-82% the years after vaccine introduction. In region Västra Götaland the yearly variation was 65-75% and in Stockholm 77-90%. These numbers were compared with vaccination coverage among the children with T1D. For the children in this study, vaccination coverage with rotavirus vaccine was 72% in region Jönköping, 80% in region Västra Götaland and 65% in region Stockholm (**Appendix 4**). This indicates that children who later developed T1D was vaccinated to the same extent as children not developing T1D.

## 5 Discussion

The main finding in this study is that rotavirus vaccination is not associated with the incidence of T1D among Swedish children or change in clinical manifestation at onset of T1D.

Concerning the rotavirus vaccination, it has been offered to infants since 2014 in Sweden which results in a maximum age of 5 years in 2019 for the children who received the vaccine first. Thus, reduction in incidence due to the rotavirus vaccine should be expected in children aged 0-4·9 years in group I but there was no such reduction. Instead, there was a significant reduction in incidence after 2014 among the oldest children in group I, who had not received rotavirus vaccination, along with the youngest and the ones aged 5-9·9 years in group III, who also were unvaccinated. This result is comparable to the reduction seen among children aged 0-4 years before and after vaccine introduction in Australia, which was considered to be an effect of the rotavirus vaccine, but with a similar size of reduction seen also in other age groups, although not statistically significant (19). In the U.S. one study found a significant reduction with 41% in T1D incidence interpreted to be due to rotavirus vaccination (20) while two other studies did not find any association (21, 22). The second study had a longer follow-up time than the first one and adjusted for potential confounding variables such as birth weight, gestation age, ethnicity, family history of T1D and breastfeeding.

The lack of association between T1D incidence and rotavirus vaccination found in our study is similar to the results of the Finnish (23) and the British (24) study but contrary to results from Australia (19) and the U.S (20). Sweden and Finland have a higher incidence of T1D compared to Australia and the U.S (5). It cannot be excluded that the effect of rotavirus vaccine varies depending on different genetic and/or environmental conditions.

Whether the vaccination may change the clinical manifestation of T1D was also investigated. Even though the incidence rate is not affected, the vaccinated children might get a less severe disease. However, this does not seem to be the case. The three groups had almost identical values for pH and HbA1c both before and after the vaccine introduction. Regarding HbA1c, all the groups actually tended to have a higher HbA1c at onset 2015-2019 than 2009-2014. Regarding pH there were no differences. Effect on clinical manifestation has not been investigated to the same extent in previous studies, why our study contributes with new information related to clinical practice.

In general, the incidence for T1D among Swedish children under the age of 15 years has not differed greatly during the observed period. However, the year of 2018 stands out compared to other years with a rate of 35·44 cases per 100 000 children, which is remarkably lower than the mean rate of 42·61 cases per 100 000 persons during these 11 years. Incomplete reporting of cases could be one reason. As seen in **Figure 2**, a decrease in incidence in 2018 was present among all age groups, specifically among the 5-9·9-year-olds and in every vaccine group. A significant increase in the total number of children in Sweden this particular year could also have had an impact on incidence rates, but as seen in **Appendix 1**, this is not the case. The total number of children in Sweden has increased, at a steady pace, during the observed years and should not affect 2018 more than the other years.

Regarding the general incidence rates in the three different vaccine groups, incidence was lower in group I compared to group II and III. Group I consisted of two regions only, including the largest one (Stockholm). Previous studies have shown that children living in big cities have a lower incidence of T1D than children living outside the cities and in the countryside (31).

### 5.1 Limitations and strengths

Since this is a register-based study, there could be errors in the reporting of the data. We were not able to identify individually which children had been vaccinated more than in certain regions, where we then found no difference in vaccination rate among those who developed or did not develop T1D. A limitation is also the rather short follow-up, as we cannot exclude that rotavirus vaccination influences the disease process with effects many years later. We will continue to follow this population and hopefully, in the future, we will be able to investigate the effect on autoantibodies related to different HLA-types and different age groups.

A strength of our study is that it includes a large sample size - all children with T1D in Sweden. Sweden has a high incidence of T1D and a high coverage of rotavirus vaccination immediately after introduction. SWEDIABKIDS is a comprehensive well validated register, which has made it possible to get reliable information of all newly-diagnosed cases of T1D parallel in time in regions with or without rotavirus vaccination.

## 6 Conclusion

We find no associations between rotavirus vaccination and the incidence of T1D, and no change in clinical manifestation at disease onset. Rotavirus vaccination is of great value, but for other reasons than prevention of T1D.

## Data availability statement

The original contributions presented in the study are included in the article/**Supplementary Material**. Further inquiries can be directed to the corresponding author.

## Ethics statement

The studies involving human participants were reviewed and approved by Swedish Ethical Review Authority. Written informed consent for participation was not provided by the participants’ legal guardians/next of kin because: The study is a register study where informed consent is not needed.

## Author contributions

AR has made most of the analyses of data and wrote the first draft of the manuscript. KÅ has contributed to creating the data base from SWEDIABKIDS and supervised the analyses of data. JL came with the concept and research idea and supervised. CO and MG contributed to the collection of data through contact with Child Head Services. All authors have critically reviewed and approved the final version of the manuscript. All authors contributed to the article and approved the submitted version.
