# Supplementary material for: No association between incidence of type 1 diabetes and rotavirus vaccination in Swedish children
Source: Front Immunol. 2023 Aug 10;14:1175071. doi: 10.3389/fimmu.2023.1175071 (PMC10456946; doi:10.3389/fimmu.2023.1175071)
Supplement: Supplementary file 1 [file Table_1.docx]

# Appendix

**Appendix 1**. Number of T1D cases and total number of children in Sweden in different age groups.

|  | **2009** | **2010** | **2011** | **2012** | **2013** | **2014** | **2015** | **2016** | **2017** | **2018** | **2019** | **N** |
| --- | --- | --- | --- | --- | --- | --- | --- | --- | --- | --- | --- | --- |
| **Sweden** |  |  |  |  |  |  |  |  |  |  |  |  |
| All cases 0-14.9 years | 686 | 669 | 697 | 724 | 710 | 754 | 732 | 767 | 748 | 645 | 761 | 7893 |
| All children 0-14.9 years | 1549442 | 1564959 | 1584270 | 1611859 | 1646101 | 1682033 | 1717143 | 1760994 | 1794677 | 1819729 | 1834821 |  |
|  |  |  |  |  |  |  |  |  |  |  |  |  |
| Cases 0-4.9 years | 170 | 170 | 170 | 166 | 170 | 171 | 153 | 161 | 146 | 130 | 177 | 1784 |
| All children 0-4.9 years | 548222 | 561788 | 567369 | 573583 | 579019 | 584157 | 586218 | 597041 | 602044 | 604498 | 601718 |  |
|  |  |  |  |  |  |  |  |  |  |  |  |  |
| Cases 5-9.9 years | 246 | 234 | 258 | 273 | 272 | 273 | 255 | 285 | 287 | 222 | 300 | 2905 |
| All children 5-9.9 years | 503934 | 515657 | 530937 | 543993 | 557950 | 571614 | 589118 | 600853 | 612180 | 619473 | 624084 |  |
|  |  |  |  |  |  |  |  |  |  |  |  |  |
| Cases 10-14.9 years | 270 | 265 | 269 | 285 | 268 | 310 | 324 | 321 | 315 | 293 | 284 | 3204 |
| All children 10-14.9 years | 497286 | 487514 | 485964 | 494283 | 509132 | 526262 | 541807 | 563100 | 580453 | 595758 | 609019 |  |

|  | **2009** | **2010** | **2011** | **2012** | **2013** | **2014** | **2015** | **2016** | **2017** | **2018** | **2019** | **N** |
| --- | --- | --- | --- | --- | --- | --- | --- | --- | --- | --- | --- | --- |
| **0-14.9 years** |  |  |  |  |  |  |  |  |  |  |  |  |
| Group I cases | 149 | 142 | 174 | 179 | 190 | 191 | 179 | 183 | 190 | 137 | 178 | 1892 |
| Group I all children | 419864 | 428538 | 437903 | 448187 | 459118 | 470102 | 479871 | 489328 | 497526 | 503739 | 507675 |  |
|  |  |  |  |  |  |  |  |  |  |  |  |  |
| Group II cases | 251 | 250 | 255 | 254 | 229 | 255 | 250 | 278 | 255 | 264 | 269 | 2810 |
| Group II all children | 515888 | 518894 | 523043 | 530728 | 541411 | 552460 | 563147 | 578056 | 588680 | 596890 | 601743 |  |
|  |  |  |  |  |  |  |  |  |  |  |  |  |
| Group III cases | 286 | 277 | 268 | 291 | 291 | 308 | 303 | 306 | 303 | 244 | 314 | 3191 |
| Group III all children | 613690 | 617527 | 623324 | 632944 | 645572 | 659471 | 674125 | 693610 | 708471 | 719100 | 725403 |  |
| **0-4.9 years** |  |  |  |  |  |  |  |  |  |  |  |  |
| Group I cases | 34 | 41 | 50 | 49 | 43 | 42 | 34 | 45 | 41 | 26 | 45 | 450 |
| Group I all children | 156573 | 161066 | 163257 | 164789 | 166428 | 167581 | 167654 | 168952 | 169746 | 169724 | 168413 |  |
|  |  |  |  |  |  |  |  |  |  |  |  |  |
| Group II cases | 68 | 58 | 56 | 51 | 60 | 54 | 49 | 54 | 42 | 52 | 63 | 607 |
| Group II all children | 178651 | 183015 | 184774 | 186564 | 188526 | 190103 | 190844 | 195015 | 196689 | 198110 | 197398 |  |
|  |  |  |  |  |  |  |  |  |  |  |  |  |
| Group III cases | 68 | 71 | 64 | 66 | 67 | 75 | 70 | 62 | 63 | 52 | 69 | 727 |
| Group III all children | 212998 | 217707 | 219338 | 222230 | 224065 | 226473 | 227720 | 233074 | 235609 | 236664 | 235907 |  |
| **5-9.9 years** |  |  |  |  |  |  |  |  |  |  |  |  |
| Group I cases | 54 | 46 | 66 | 56 | 74 | 65 | 67 | 73 | 75 | 45 | 74 | 695 |
| Group I all children | 137181 | 142215 | 147985 | 152596 | 156831 | 160372 | 164747 | 167126 | 169472 | 171018 | 172657 |  |
|  |  |  |  |  |  |  |  |  |  |  |  |  |
| Group II cases | 85 | 94 | 87 | 97 | 78 | 95 | 87 | 101 | 108 | 100 | 99 | 1031 |
| Group II all children | 166719 | 169467 | 173387 | 177751 | 182224 | 187153 | 193010 | 197231 | 200620 | 203091 | 204276 |  |
|  |  |  |  |  |  |  |  |  |  |  |  |  |
| Group III cases | 107 | 94 | 105 | 120 | 120 | 113 | 101 | 111 | 104 | 77 | 127 | 1179 |
| Group III all children | 214019 | 218098 | 223913 | 228223 | 233684 | 239137 | 246797 | 252076 | 257890 | 261417 | 263278 |  |
| **10-14.9 years** |  |  |  |  |  |  |  |  |  |  |  |  |
| Group I cases | 61 | 55 | 58 | 74 | 73 | 84 | 78 | 65 | 74 | 66 | 59 | 747 |
| Group I all children | 126110 | 125257 | 126661 | 130802 | 135859 | 142149 | 147470 | 153250 | 158308 | 162997 | 166605 |  |
|  |  |  |  |  |  |  |  |  |  |  |  |  |
| Group II cases | 98 | 98 | 112 | 106 | 91 | 106 | 114 | 123 | 105 | 112 | 107 | 1172 |
| Group II all children | 170518 | 166412 | 164882 | 166413 | 170661 | 175204 | 179293 | 185810 | 191371 | 195689 | 200069 |  |
|  |  |  |  |  |  |  |  |  |  |  |  |  |
| Group III cases | 111 | 112 | 99 | 105 | 104 | 120 | 132 | 133 | 136 | 115 | 118 | 1285 |
| Group III all children | 200658 | 195845 | 194421 | 197068 | 202612 | 208909 | 215044 | 224040 | 230774 | 237072 | 242345 |  |

**Appendix 2.** Number of T1D cases and total number of children in the three vaccination groups and age groups.

*Appendix 2. Group I started rotavirus vaccination in 2014, group II started sometime between 2016-2018 and group III started in 2019.*

**Appendix 3.** List of included counties and number of children with T1D in each vaccination group

| Group I (N=1892) | Group II (N=2810) | Group III (N=3191) |
| --- | --- | --- |
| Jönköping | Dalarna | Blekinge |
| Stockholm | Södermanland | Gotland |
|  | Värmland | Halland |
|  | Västerbotten | Jämtland |
|  | Västra Götaland | Kalmar |
|  | Örebro  Gävleborg | Kronoberg  Norrbotten |
|  |  | Skåne |
|  |  | Uppsala |
|  |  | Västmanland |
|  |  | Västernorrland |
|  |  | Östergötland |
|  |  |  |
|  |  |  |

*Appendix 3. Group I started rotavirus vaccination in 2014, group II started sometime between 2016 and 2018 and group III started in 2019.*

**Appendix 4.** Number and proportion of rotavirus vaccinated children with T1D

|  | Proportion of vaccinated T1D children (%) | Children offered vaccine (n) | Children receiving vaccine (n) | Regional overall coverage the years after vaccine introduction (%) |
| --- | --- | --- | --- | --- |
| **Region Jönköping** | 72% | 29 | 21 | 76-82% |
| **Region Stockholm** | 69% | 65 | 45 | 77-90% |
| **Region Västra Götaland** | 81% | 31 | 25 | 65-75% |
| **Total** | 73% | 125 | 91 |  |

*Appendix 4. Region Jönköping and Stockholm introduced the rotavirus vaccine in 2014 and region Västra Götaland in 2016.*

**Appendix 5.** Number of children with T1D in age groups.

| **Age** | **Boys** | **Girls** | **Total** |
| --- | --- | --- | --- |
| 0-14·9 years (100) | 4252 (53·9) | 3641 (46·1) | 7893 (100) |
| 0-4·9 years (22·6) | 967(54·2) | 817 (45·8) | 1784 (100) |
| 5-9·9 years (36·8) | 1470 (50·6) | 1435 (49·4) | 2905 (100) |
| 10-14·9 years (40·6) | 1815 (56·6) | 1389 (43·4) | 3204 (100) |
